# Autoantibodies neutralizing GM-CSF in HIV-negative Colombian patients infected with Cryptococcus gattii and C. neoformans

**DOI:** 10.21203/rs.3.rs-3873029/v1

**Published:** 2024-01-19

**Authors:** Carlos A. Arango-Franco, Julián Rojas, Carolina Firacative, Clara Inés Agudelo, José Luis Franco, Jean-Laurent Casanova, Anne Puel, Jairo Lizarazo, Elizabeth Castañeda, Andrés A. Arias

**Affiliations:** University of Antioquia (UdeA); University of Antioquia (UdeA); University del Rosario; Instituto Nacional de Salud; University of Antioquia (UdeA); INSERM U1163, Necker Hospital for Sick Children; INSERM U1163, Necker Hospital for Sick Children; University of Pamplona; Instituto Nacional de Salud; University of Antioquia (UdeA)

**Keywords:** Cryptococcosis, Granulocyte-Macrophage Colony-Stimulating Factor (GM-CSF), Cryptococcus neoformans, Cryptococcus gattii, GM-CSF autoantibodies, anti-cytokine autoantibodies, human immunodeficiency virus (HIV), Mycobacterium tuberculosis (Mtb), Pulmonary tuberculosis (Tb), autoantibodies (auto-Abs) neutralizing GM-CSF, HIV-negative patients

## Abstract

**Background:**

Cryptococcosis is a life-threatening disease caused by *Cryptococcus neoformans* or *C. gattii*. Autoantibodies (auto-Abs) neutralizing granulocyte-macrophage colony-stimulating factor (GM-CSF) in otherwise healthy adults with cryptococcal meningitis have been described since 2013. We searched for neutralizing auto-Abs in sera from Colombian patients with non-HIV related cryptococcosis in a retrospective national cohort collected from 1997 to 2016.

**Methods:**

We reviewed clinical and laboratory records and assessed the presence of neutralizing auto-Abs in 30 HIV (−) adults presenting cryptococcosis (13 by *C. gattii*, and 17 by *C. neoformans*).

**Results:**

We detected auto-Abs neutralizing GM-CSF in the plasma of 9 out of 13 (69%) patients infected with *C. gattii* and 1 out of 17 (6%) patients with *C. neoformans*.

**Conclusions:**

We report ten Colombian patients with cryptococcosis due to auto-Abs neutralizing GM-CSF. Nine of the ten patients were infected with *C. gattii*, and only one with *C. neoformans*.

## Introduction

Cryptococcosis is initiated by breathing blastoconidium or basidiospores of the yeasts of the species complexes *Cryptococcus neoformans* and *C. gattii* [1]. After inhaling the propagules from the environment, mainly soil, avian excreta, trees, and decaying wood, cryptococcal infection presents initially as pneumonia, to later disseminate to the central nervous system (CNS), causing meningitis, the most frequent form, or even meningoencephalitis [1].

Despite common environmental exposure to cryptococcal species, cryptococcosis is rare in the healthy population because of high natural resistance. Defects in T cell-mediated immunity, specifically the decline in number and function of CD4^+^ lymphocytes, which usually occurs in people infected with the human immunodeficiency virus (HIV), remains the main risk factor to acquire infection by *C. neoformans* [1]. Cryptococcosis caused by *C. gattii*, which is much less common (~ 20%), has traditionally been associated in otherwise healthy individuals, particularly HIV-seronegative, or those with unidentified risk factors [2]. However, other immunosuppressive and pulmonary diseases are underlying conditions that have been associated with a significant higher risk of *C. gattii* infection [2]. Moreover, in the last decade, subtle immunologic alterations, which are host-dependent risk factors, have been detected in most patients with cryptococcosis by *C. gattii*, placing this species as an opportunistic pathogen [3, 4].

Anti-cytokine neutralizing autoantibodies (auto-Abs) are considered to constitute autoimmune phenocopies of Inborn Error of Immunity (IEI) with selective predisposition to infectious diseases [5–7]. Indeed, by blocking the biological function of their target cytokines, these auto-Abs cause clinical phenotypes mimicking those of IEI of the corresponding cytokines or their receptors [7]. Patients affected by different infectious diseases, including fungal infections, have been found to carry such neutralizing auto-Abs [5]. For example, chronic mucocutaneous candidiasis is attributed to interleukin (IL)-17A/F auto-Abs, adult-onset susceptibility to mycobacterial disease to interferon-gamma (IFN-γ) auto-Abs, recurrent staphylococcal infection to IL-6 auto-Abs, and more recently, severe SARS-CoV2 infection to type I IFNs auto-Abs [5, 7].

Since 2013, high titers of immunoglobulin G (IgG) auto-Abs neutralizing granulocyte-macrophage colony-stimulating factor (GM-CSF) have been identified in patients with adult-onset isolated idiopathic disseminated diseases, mostly cryptococcosis, almost exclusively by *C. gattii* [8–10], and other infections such those caused by species of *Nocardia* and more rarely *Aspergillus* [9, 10]. In addition to the infectious phenotype, those auto-Abs against GM-CSF have also been associated with pulmonary alveolar proteinosis (PAP), a severe lung disease characterized by the accumulation of surfactant in the alveoli, with progressive respiratory failure and an increased risk of secondary infections [11]. The causality among the presence of neutralizing auto-Abs GM-CSF and these two clinical phenotypes (PAP and cryptococcosis) is not well understood. Nevertheless, patients with such auto-Abs presenting with cryptococcosis have been reported with or without PAP manifestations, and patients with PAP have been described with or without cryptococcosis [8]. Finally, the presence of auto-Abs against GM-CSF in those pathologies, suggests an important role of GM-CSF in the correct maturation and function of alveolar macrophages, which constitutes the main cellular component of immunity against *Cryptococcus* [12, 13].

The fact that about 13% of cases of cryptococcosis in Colombia occur in non-HIV patients with no apparent risk factor [14, 15], together with the recent identification of auto-Abs neutralizing GM-CSF in three Colombian patients with cryptococcal meningitis by *Cryptococcus* spp. [16], prompted us to hypothesize that other Colombian patients affected by this mycosis and who had been regarded as otherwise healthy individuals, especially given that they were not infected with HIV, have a hidden immune dysfunction. As such, the aim of this study was to establish the presence of auto-Abs against GM-CSF in plasma or serum from 30 Colombian patients, without HIV, who developed cryptococcosis by *C. gattii* or *C. neoformans* species complexes, and to correlate our findings with the patients’ clinical data.

## Materials and methods

### Subjects and sera selection

As part of the National Surveillance Program for *Cryptococcus* and cryptococcosis in Colombia, led by the Instituto Nacional de Salud, in Bogota, Colombia, 1974 surveys from patients with cryptococcosis have been filled out between 1997 and 2016. In summary, the survey contains demographic data, risk factors, clinical manifestations, diagnostic methods, and the patients’ initial treatment [14]. From these surveys, 392 (19.9%) patients were reported without evident HIV infection (HIV negative), from whom, the etiological agent causing cryptococcosis was identified in 343 cases, 292 (85.1%) caused by *C. neoformans* and 51 caused by *C. gattii* (14.9%) [14] **(See supplementary Annex 1).** From all non-HIV-related cryptococcosis, only 30 patients’ serum samples were collected in a 15-year period, from 1997 to 2011, and stored in the sera collection of the Microbiology Group at the Instituto Nacional de Salud. From these available samples, which were used to establish the presence of GM-CSF auto-Abs, 13 sera were from patients with cryptococcosis by *C. gattii* and 17 sera by *C. neoformans* [14] **(See in supplementary Tables S1, S2 and S3).**

As part of the diagnosis of cryptococcosis, which was done by direct visualization of the encapsulated blastoconidia in cerebrospinal fluid (CSF) using India ink, some of the studied sera had data on the titter of the cryptococcal antigen (CrAg) detected in this sample. CrAg titter was also established in CSF of some patients. All isolates of *C. gattii* or *C. neoformans* causing cryptococcosis in the studied patients were identified by routinely used phenotypic methods. Most *C. gattii* isolates had data on antifungal susceptibility testing, serotype, mating type, molecular type and sequence type (ST), while most *C. neoformans* isolates had only data on serotype, mating type and molecular type [14, 15, 17] **(Tables S4 and S5).** All studied sera had in addition total levels of IgG, IgA and IgM, as well as cryptococcal-specific IgG, IgA and IgM, as established previously [18]. For some analysis, data on specific levels of serum immunoglobulins against cryptococcal proteins were used from serum from adults without cryptococcosis or any other infectious disease (healthy controls) [18] **(See in supplementary Figures S1 and S2 ).**

### Detection of neutralizing GM-CSF auto-Abs by flow cytometry

Human peripheral blood mononuclear cells (PBMCs) from a healthy donor were isolated from whole blood by Ficoll- Hypaque density centrifugation (Amersham-Pharmacia- Biotech). The cells were counted and plated at 2 × 10^6^ cells/well in 96-well V-bottom plates (Thermo-Fisher-Scientific), in 100 μL of RPMI (GibcoBRL, Invitrogen) supplemented with 10% fetal bovine serum (GibcoBRL, Invitrogen), or 100 μL of RPMI supplemented with 1:10 serafrom patients or controls. PBMCs were left unstimulated or were stimulated with 10 ng/μL of rhIL-3 or GM-CSF or 50 ng/μL of rhIL-3 (Miltenyi-Biotec) for 15 min at 37°C. Thereafter, cells were fixed and permeabilized with a fixation/permeabilization kit (eBioscience). Extracellular labeling was performed with antibodies anti CD14-Pacific Blue and anti CD4-FITC (Sony-Biotechnology, clones M5E2 and RPA-T4, respectively). Cell viability was determined with the Aqua Dead Cell Stain Kit (Thermo-Fisher-Scientific). STAT5 phosphorylation (p-STAT5) levels were assessed by intracellular staining with Phospho-Flow PE Mouse Anti-p-STAT5 (pY694) antibody (BD Biosciences). Data were collected with a Gallios flow cytometer (Beckman-Coulter) and analyzed with FlowJo software v.10.6.2 (Becton–Dickinson).

### Statistical analysis and data availability

p-value was calculated among groups with a Chi-square test with Fisher’s correction (given the low n in some of the cells of the contingency tables). A p-value < 0.05 was considered as statistically significant. All raw and processed data will be made available by the corresponding authors upon request.

## Results

### Cryptococcosis in Colombian HIV negative patients

Demographic and clinical characteristics of the 30 studied patients are summarized in the supplementary material **(Supplementary Tables 1, 2 and 3).** From them, 21 (70%) were men and nine (30%) were women, ranging from 1 to 71 years old, with an average age of 40.8 years. While most patients (86.7%) did not have any recognizable predisposing factor, hematological malignancy was registered in two patients (6.7%) as well as systemic lupus erythematosus (SLE) and rheumatoid arthritis in one patient each (3.3%). Signs and symptoms of cryptococcosis were variable among the studied patients, however, headache (70%), mental confusion (46.7%) and nausea (40%) were the most frequent clinical manifestations, considering that most patients (96.7%) were diagnosed with cryptococcal meningitis. Treatment was mainly amphotericin B deoxycholate alone (46.7%), or in combination with fluconazole (30%). Outcome of patients was recorded in 11 cases, from whom eight recovered after treatment while three died of cryptococcosis [14]

### Detection of neutralizing GM-CSF auto Abs in patients’ sera

**Figure 1:**
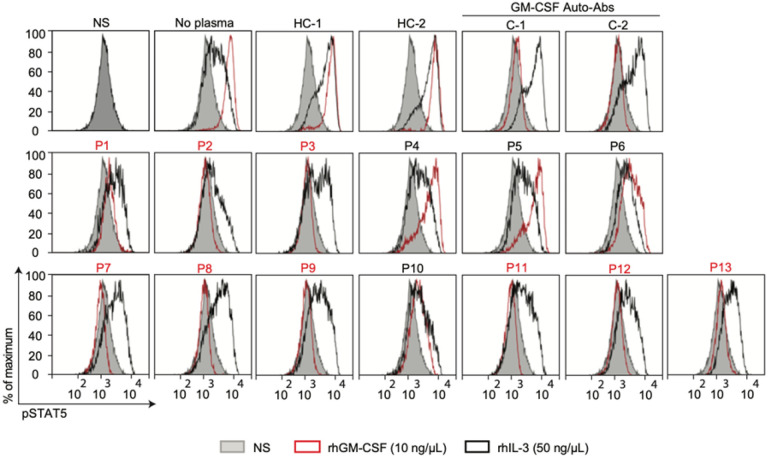
Neutralizing GM-CSF auto-Abs in patients with cryptococcosis by Cryptococcus gattii. STAT5 phosphorylation (p-STAT5), assessed by flow cytometry, upon the stimulation with recombinant human rhGM-CSF (red) or rhIL-3 (black) of control PBMCs, in the absence of sera, or in the presence of 1:10 dilution of sera from two healthy individuals (HC-1 and HC-2), sera of two individuals previously described carrying GM-CSF auto-Abs (C-1 and C-2) or from thirteen patients with cryptococcosis by *C. gattii*. NS: non-stimulated with rhGM-CSF or rhIL-3.

**Figure 2:**
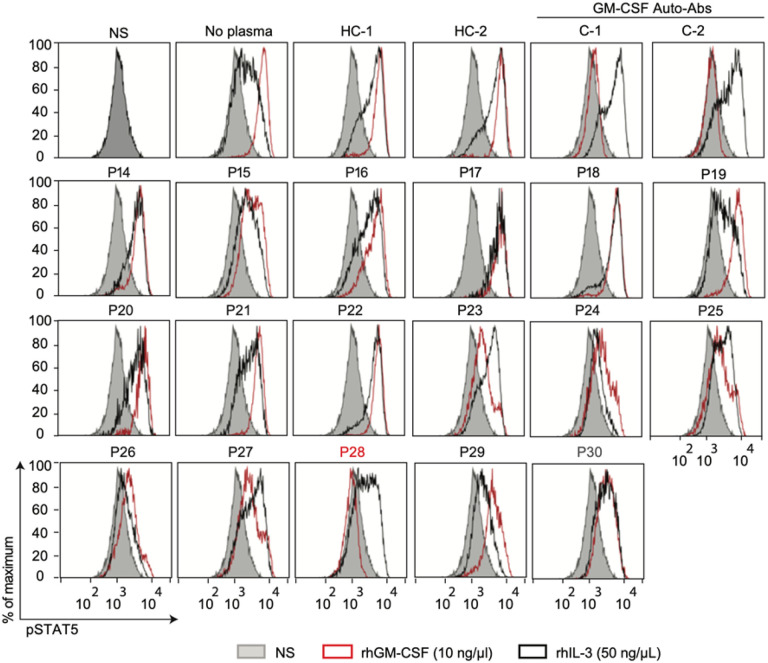
Neutralizing GM-CSF auto-Abs in patients with cryptococcosis by Cryptococcus neoformans. STAT5 phosphorylation (p-STAT5), assessed by flow cytometry, upon the stimulation with recombinant human rhGM-CSF (red) or rhIL-3 (black) of control PBMCs, in the absence of sera, or in the presence of a 1:10 dilution of serum from two healthy individuals (HC-1 and HC-2), sera of two individuals previously described carrying GM-CSF auto-Abs (C-1 and C-2) or from seventeen patients with cryptococcosis by *C. neoformans*. NS: non-stimulated with rhGM-CSF or rhIL-3.

First, we evaluated the *C. gattii* patient’s cohort; unlike serum from healthy individuals, serum from nine patients (69.2%), incubated with 10 ng/μL of GM-CSF prevented GM-CSF-induced p-STAT5, whereas the level of IL-3-induced p-STAT5 was similar in cells incubated with controls or patients’ sera (Fig. 1). Regarding the *C. neoformans* cohort, one out of 17 s patients (6%) presented neutralizing GM-CSF auto-Abs **(**Fig. 2**)**. Altogether, these results strongly suggest that the presence of circulating neutralizing GM-CSF auto-Abs is the risk factor to develop cryptococcosis in 10 of the studied patients.

### Clinical and immunological correlation in patients with neutralizing GM-CSF auto-Abs

From the 30 studied patients, nine (69.2%) and one (6%) patient affected with cryptococcosis by *C. gattii* and *C. neoformans*, respectively, were positive for neutralizing GM-CSF auto-Abs. The demographic, clinical and microbiological characteristics of both groups (positive and negative for neutralizing GM-CSF auto-Abs) are compared (Table 1**)**,. Clearly, patients with neutralizing GM-CSF auto-Abs are predominantly affected by *C. gattii*. Sex, age, and clinical presentation did not differ among patients with and without neutralizing GM-CSF auto-Abs. Clinical characteristics of patients with cryptococcosis with and without neutralizing GM-CSF auto-Abs respectively, are summarized in **Supplementary Tables 2–3.** Total and specific levels of immunoglobulins were determined, however total levels of IgG, IgM and IgA did not differ between patients with or without neutralizing GM-CSF auto-Abs, nor if they were infected by *C. neoformans* or *C. gattii* (**Supplementary Fig. 1a–c**). When compared with healthy controls, we observed higher levels of total IgG in patients with cryptococcosis, whereas total IgA and IgM were in lower levels. Nevertheless, sera from patients with cryptococcosis by *C. gattii* with neutralizing GM-CSF auto-Abs showed higher levels of specific IgG against cryptococcal proteins, compared to healthy controls, as established elsewhere (**Supplementary Figs. 1d–e**) [18].

### Phenotypic and genotypic characteristics of cryptococcal isolates do not differ among patients with and without neutralizing GM-CSF auto-Abs

All *C. gattii* isolates causing cryptococcosis in patients with and without neutralizing GM-CSF auto-Abs, which had data on antifungal susceptibility testing, distributed among the wild-type population of the species, per antifungal drug (**Supplementary Tables 4 and 5**). This means that none of them showed resistance or decreased susceptibility to any antifungal drug. In addition, *C. gattii* isolates recovered from patients with and without neutralizing anti GM-CSF auto-Abs, did not differ regarding the serotype, mating type, molecular type, and ST of the isolates (**Supplementary Fig. 1f**). Serotype, mating type and molecular type did not differ either among the *C. neoformans* isolates.

## Discussion

Our study reports neutralizing GM-CSF auto-Abs in 10 Colombian patients who developed cryptococcosis, even though these patients were deemed as otherwise healthy, based upon their clinical history at the time of diagnosis of this mycosis [14]. We also evidence that this subtle immunologic alteration prevails in patients affected by *C. gattii*, compared with patients affected by *C. neoformans*, which adds to the differences described in the epidemiology of patients with cryptococcosis caused by one species complex or another [3, 4]. Remarkably, the risk factor of patients with *C. gattii* infection and 16 with *C. neoformans* infection, remains hidden, as neutralizing auto-Abs against GM-CSF were not detected.

The epidemiology of cryptococcosis has focused especially on patients with HIV, the major risk factor for infection [19]. Though, in recent years, different studies on cryptococcosis have drawn attention to the increase in those apparently otherwise healthy individuals without HIV infection [20–23]. In Colombia, in the cryptococcoses survey performed between 1997–2016, HIV infection was found in 75.4% of patients and non-HIV in 24.6% [14]. In Brazil, HIV infection remains the main risk factor for cryptococcosis (82–86%) [24, 25], however, in a series of 29 patients without HIV infection and who did not receive transplants, 77.8% of the patients had no apparent risk factor and the majority were infected by *C. gattii* [21]. Similarly, in the French Guiana, HIV infection has been reported as the main risk factor for cryptococcosis (67.4%), but it was precisely in this country where was reported the first two cases of Latin-American patients with cryptococcosis presenting anti-GM-CSF auto-Abs in otherwise healthy individuals with *C. gattii* infection [26].These observations indicate that the epidemiology of cryptococcosis is changing with a notable increase of non-HIV-related infection suggesting that additional risk factors may be involved in the susceptibility to cryptococcosis.

In Colombia, 1974 patients with cryptococcosis have been identified between 1997 and 2016. From them, just 392 patients were HIV-negative, of whom 51 (14.9%) were affected by *C. gattii* infection. The study of 13 patients with *C. gattii* and 17 with *C. neoformans* from this cohort allows us to establish that those auto-Abs collectively account for 68% (n = 9) of *C. gattii* patients and 6% (n = 1) of *C. neoformans*, showing that GM auto-Abs underlie cryptococcosis in a significant proportion of cases. Nevertheless, cryptococcosis remains unexplained in most HIV-negative patients affected with *C. neoformans* and one-third of *C. gattii*. IEI of GM-CSF pathway cytokine or their receptors might potentially explain a proportion of these remaining cases. Similarly, to previous studies, all 10 patients with GM-CSF auto-Abs described here were adults (23 to 67 years old). If those auto-Abs were present before cryptococcal infection and if they remained silent up to cryptococcal infection, remains unknown. Recent studies of auto-Abs against cytokines as a main risk factor for a specific infectious disease have demonstrated the causality of those auto-Abs [27–29]. Pre-existing Type I IFN neutralizing auto-Abs is the main clear example as a risk factor for several viral diseases such as life-threatening COVID-19 pneumonia (15–20%), a third of the rare life-threatening adverse reactions to yellow fever vaccination [28], and about 40% of cases of West Nile virus encephalitis [30]. In addition, these auto-Abs showed to be present in around ~0.3% of the general population under 65 years, whereas this prevalence increases sharply after 70 years to ~4% [28–30]. The actual prevalence of GM-CSF auto-Abs in patients with cryptococcosis or healthy populations remains elusive.

Interestingly, one patient described here (P28) presented disseminated cryptococcosis by *C. neoformans* and pulmonary tuberculosis (Tb) by Mycobacterium tuberculosis (Mtb) one year after the cryptococcal diagnosis. To our knowledge, this is the third adult patient with disseminated cryptococcosis and neutralizing anti-GM-CSF auto-Abs who have developed Tb [8, 16]. Furthermore, mice studies and *ex vivo* studies, and human monocyte-derived macrophages [31–33], and our date suggest that intact GM-CSF signaling is crucial for the appropriate alveolar macrophage functions to mediate the immunity to Cryptococcus spp., and possibly against *Mtb* lung infection in humans.

Our findings support the hypothesis, and the new research front, that auto-Abs against cytokines may explain the susceptibility to Cryptococcus infection in otherwise healthy HIV-negative individuals [7]. However, routinely used laboratory technologies do not detect such specific targets [16, 34, 35], and ow cytometry and particle-based technology used to screen for the presence of anti-cytokines auto-Abs, are very expensive, not often available in the clinical setting and, so far, have been utilized as research tools rather than as commonly used techniques to help establishing a definitive diagnosis in patients with Infectious diseases, like cryptococcosis [8, 9, 16, 36].

## Conclusions

Our findings, therefore, support the association between neutralizing GM-CSF auto-Abs and cryptococcosis, which has been described for a decade [34–36]. These auto-Ab can be totally silent until the patients are diagnosed with cryptococcosis. However, the reason why patients with this predisposing factor are more susceptible to acquire infection by *C. gattii* than by *C. neoformans*, remains elusive. Anti-GM-CSF auto-Abs are associated with some cases of pulmonary and meningeal cryptococcosis in otherwise healthy individuals. Our data support the idea that GM-CSF is a critical actor in host defense against *Cryptococcus*.

## Figures and Tables

**Table 1:** Characteristics of patients with cryptococcosis with (+) and without (−) GM-CSF auto-Abs

Demographic, clinical, and microbiological variables	GM-CSF auto-Abs (+) (n = 10)	GM-CSF auto-Abs (−) (n = 20)	*p*-value^1^
Male/Female ratio	7:3	14:6	1
Male	70%	70%	1
Age: mean - extreme values (years)	38.5 (23–67)	40.45 (1–71)	
Clinical presentation			
CNS involvement	8 (80%)	18 (90%)	0.5837
Lung involvement	1 (10%)	2 (10%)	1
CNS + lung involvement^2^	1 (10%)	0	0.3293
Risk factor non-HIV			
Unknown	10 (100%)	15 (75%)	0.1394
Hematological malignancy	0	2 (10%)	0.5477
Autoimmune disease (SLE, RA)^3^	0	2 (10%)	0.5477
Solid neoplasm	0	1	1
Treatment			
AMBd + 5 FC^4^	2 (20%)	0	0.09145
AMBd + FLC^4^	5 (50%)	3 (15%)	0.07396
AMBd^4^	2 (20%)	12 (60%)	0.05397
No data	1 (10%)	5 (25%)	0.6582
Outcome			
Cure	6 (60%)	2 (10%)	0.005997
Deceased	1 (10%)	2 (10%)	1
No data	3 (30%)	16 (80%)	0.01799
Proven cryptococcosis			
Culture	10 (100%)	20 (100%)	NA
Species complex			
*C. gattii*	9 (90%)	4 (20%)	0.0009995
*C. neoformans*	1 (10%)	16 (80%)	0.0004998
Molecular types			
VNI	1 (10%)	16 (80%)	0.0004998
VGI	1 (10%)	0	0.3293
VGII	7 (70%)	2 (10%)	0.002499
VGIII	1 (10%)	2 (10%)	1
Serotype			
- A	1 (10%)	16 (80%)	0.0004998
- B	9 (90%)	3 (15%)	0.0004998
- C	0	1 (5%)	1
- D	0	0	NA

1 (p-value calculated with a Chi-square test with Fisher’s correction given the low n in some of the cells of the contingency tables)

2 Pulmonary cryptococcoma with positive culture and histopathology

3 SLE=systemic lupus erythematosus, RA = rheumatoid arthritis

3 AMBd= amphotericin B deoxycholate, 5FC = 5-flucytosine, FLC = fluconazole

## Key Points

The importance of anti-cytokine auto-antibodies in the pathogenesis of various infectious diseases, including opportunistic mycoses is increasingly being reported.

GM-CSF auto-Abs are present in Colombian patients suffering cryptococcosis by *C. gattii* and *C. neoformans*.

## References

[1] Kwon-Chung KJ, Cryptococcus neoformans and Cryptococcus gattii, the etiologic agents of cryptococcosis. Cold Spring Harb Perspect Med. 2014;4(7):a019760. 24985132 10.1101/cshperspect.a019760 PMC4066639

[2] MacDougall L, Risk factors for Cryptococcus gattii infection, British Columbia, Canada. Emerg Infect Dis. 2011;17(2):193–9. 21291588 10.3201/eid1702.101020 PMC3204768

[3] Kwon-Chung KJ, Saijo T. Is J Fungi (Basel). 2015;1(2):154–67. 10.3390/jof1020154 PMC5084617 27795955

[4] Chen SC, Meyer W, Sorrell TC. Cryptococcus gattii infections. Clin Microbiol Rev. 2014;27(4):980–1024. 25278580 10.1128/CMR.00126-13 PMC4187630

[5] Puel A Human autoantibodies underlying infectious diseases. J Exp Med, 2022. 219(4). 10.1084/jem.20211387 PMC8952682 35319722

[6] Ku CL, Autoantibodies against cytokines: phenocopies of primary immunodeficiencies? Hum Genet. 2020;139(6–7):783–94. 32419033 10.1007/s00439-020-02180-0 PMC7272486

[7] Vinh DC. Of Mycelium and Men: Inherent Human Susceptibility to Fungal Diseases. Pathogens, 2023. 12(3). 10.3390/pathogens12030456 PMC10058615 36986378

[8] Rosen LB, Anti-GM-CSF autoantibodies in patients with cryptococcal meningitis. J Immunol. 2013;190(8):3959–66. 23509356 10.4049/jimmunol.1202526 PMC3675663

[9] Salvator H, Neutralizing GM-CSF autoantibodies in pulmonary alveolar proteinosis, cryptococcal meningitis and severe nocardiosis. Respir Res. 2022;23(1):280. 36221098 10.1186/s12931-022-02103-9 PMC9552154

[10] Lee E, Opportunistic Infection Associated With Elevated GM-CSF Autoantibodies: A Case Series and Review of the Literature. Open Forum Infect Dis. 2022;9(5):ofac146. 35531378 10.1093/ofid/ofac146 PMC9070348

[11] Trapnell BC, Pulmonary alveolar proteinosis. Nat Rev Dis Primers. 2019;5(1):16. 30846703 10.1038/s41572-019-0066-3

[12] Chen GH, Local GM-CSF-Dependent Differentiation and Activation of Pulmonary Dendritic Cells and Macrophages Protect against Progressive Cryptococcal Lung Infection in Mice. J Immunol. 2016;196(4):1810–21. 26755822 10.4049/jimmunol.1501512 PMC4744503

[13] Prevel R, Central Nervous System Cryptococcosis in Patients With Sarcoidosis: Comparison With Non-sarcoidosis Patients and Review of Potential Pathophysiological Mechanisms. Front Med (Lausanne). 2022;9:836886. 35425769 10.3389/fmed.2022.836886 PMC9002233

[14] Escandon P Cryptococcosis in Colombia: Compilation and Analysis of Data from Laboratory-Based Surveillance. J Fungi (Basel), 2018. 4(1). 10.3390/jof4010032 PMC5872335 29494502

[15] Lizarazo J, Retrospective study of the epidemiology and clinical manifestations of Cryptococcus gattii infections in Colombia from 1997–2011. PLoS Negl Trop Dis. 2014;8(11):e3272. 25411779 10.1371/journal.pntd.0003272 PMC4238989

[16] Arango-Franco CA, Anti-GM-CSF Neutralizing Autoantibodies in Colombian Patients with Disseminated Cryptococcosis. J Clin Immunol. 2023;43(5):921–32. 36821021 10.1007/s10875-023-01451-5 PMC9947894

[17] Firacative C, Escandón P. Antifungal susceptibility of clinical Cryptococcus gattii isolates from Colombia varies among molecular types. Med Mycol. 2021;59(11):1122–5. 34264298 10.1093/mmy/myab041 PMC8757315

[18] Becerra-Álvarez P Cryptococcus neoformans- and Cryptococcus gattii-specific IgG, IgA and IgM differ among children and adults with and without cryptococcosis from Colombia. Med Mycol, 2022. 60(9). 10.1093/mmy/myac067 36066645

[19] Rajasingham R, The global burden of HIV-associated cryptococcal infection in adults in 2020: a modelling analysis. Lancet Infect Dis. 2022;22(12):1748–55. 36049486 10.1016/S1473-3099(22)00499-6 PMC9701154

[20] Coussement J, Current Epidemiology and Clinical Features of Cryptococcus Infection in Patients Without Human Immunodeficiency Virus: A Multicenter Study in 46 Hospitals in Australia and New Zealand. Clin Infect Dis. 2023;77(7):976–86. 37235212 10.1093/cid/ciad321

[21] Brizendine KD, Baddley JW, Pappas PG. Predictors of mortality and differences in clinical features among patients with Cryptococcosis according to immune status. PLoS ONE. 2013;8(3):e60431. 23555970 10.1371/journal.pone.0060431 PMC3608592

[22] Hevey MA, Presentation and Mortality of Cryptococcal Infection Varies by Predisposing Illness: A Retrospective Cohort Study. Am J Med. 2019;132(8):977–983e1. 31077652 10.1016/j.amjmed.2019.04.026 PMC6744315

[23] Fang W, Fa Z, Liao W. Epidemiology of Cryptococcus and cryptococcosis in China. Fungal Genet Biol. 2015;78:7–15. 25445309 10.1016/j.fgb.2014.10.017

[24] de Azambuja AZ Cryptococcal Meningitis: A Retrospective Cohort of a Brazilian Reference Hospital in the Post-HAART Era of Universal Access. Can J Infect Dis Med Microbiol, 2018. 2018: p. 6512468. 30154942 10.1155/2018/6512468 PMC6093042

[25] Nunes JO, Cryptococcal meningitis epidemiology: 17 years of experience in a State of the Brazilian Pantanal. Rev Soc Bras Med Trop. 2018;51(4):485–92. 30133632 10.1590/0037-8682-0050-2018

[26] Debourgogne A, Characteristics and specificities of Cryptococcus infections in French Guiana, 1998–2008. Med Mycol. 2011;49(8):864–71. 21612563 10.3109/13693786.2011.584198

[27] Bastard P Autoantibodies against type I IFNs in patients with life-threatening COVID-19. Science, 2020. 370(6515). 10.1126/science.abd4585 PMC7857397 32972996

[28] Bastard P Auto-antibodies to type I IFNs can underlie adverse reactions to yellow fever live attenuated vaccine. J Exp Med, 2021. 218(4). 10.1084/jem.20202486 PMC7871457 33544838

[29] Asano T X-linked recessive TLR7 deficiency in ~ 1% of men under 60 years old with life-threatening COVID-19. Sci Immunol, 2021. 6(62). 10.1126/sciimmunol.abl4348 PMC8532080 34413140

[30] Le Voyer T, Autoantibodies against type I IFNs in humans with alternative NF-κB pathway deficiency. Nature. 2023;623(7988):803–13. 37938781 10.1038/s41586-023-06717-x PMC10665196

[31] Bryson BD, Heterogeneous GM-CSF signaling in macrophages is associated with control of Mycobacterium tuberculosis. Nat Commun. 2019;10(1):2329. 31133636 10.1038/s41467-019-10065-8 PMC6536549

[32] Rothchild AC Role of Granulocyte-Macrophage Colony-Stimulating Factor Production by T Cells during. mBio, 2017. 8(5). 10.1128/mBio.01514-17 PMC5654932 29066547

[33] Gonzalez-Juarrero M, Disruption of granulocyte macrophage-colony stimulating factor production in the lungs severely affects the ability of mice to control Mycobacterium tuberculosis infection. J Leukoc Biol. 2005;77(6):914–22. 15767289 10.1189/jlb.1204723

[34] Kuo PH, Neutralizing Anti-Granulocyte-Macrophage Colony-Stimulating Factor Autoantibodies in Patients With Central Nervous System and Localized Cryptococcosis: Longitudinal Follow-up and Literature Review. Clin Infect Dis. 2022;75(2):278–87. 34718451 10.1093/cid/ciab920

[35] Wang SY, Cryptococcus gattii Infection as the Major Clinical Manifestation in Patients with Autoantibodies Against Granulocyte-Macrophage Colony-Stimulating Factor. J Clin Immunol. 2022;42(8):1730–41. 35947322 10.1007/s10875-022-01341-2

[36] Yang DH, Cryptococcus gattii Species Complex as an Opportunistic Pathogen: Underlying Medical Conditions Associated with the Infection. mBio. 2021;12(5):e0270821. 34700378 10.1128/mBio.02708-21 PMC8546560

